# Tomographic and multimodal scattering-type scanning near-field optical microscopy with peak force tapping mode

**DOI:** 10.1038/s41467-018-04403-5

**Published:** 2018-05-21

**Authors:** Haomin Wang, Le Wang, Devon S. Jakob, Xiaoji G. Xu

**Affiliations:** Department of Chemistry, Lehigh University, 6 E Packer Avenue, Bethlehem, PA 18015 USA

## Abstract

Scattering-type scanning near-field optical microscopy (s-SNOM) enables nanoscale spectroscopic imaging and has been instrumental for many nano-photonic discoveries and in situ studies. However, conventional s-SNOM techniques with atomic force microscopy tapping mode operation and lock-in detections do not provide direct tomographic information with explicit tip−sample distance. Here, we present a non-traditional s-SNOM technique, named peak force scattering-type scanning near-field optical microscopy (PF-SNOM), by combination of peak force tapping mode and time-gated light detection. PF-SNOM enables direct sectioning of vertical near-field signals from a sample surface for both three-dimensional near-field imaging and spectroscopic analysis. Tip-induced relaxation of surface phonon polaritons are revealed and modeled by considering tip damping. PF-SNOM also delivers a spatial resolution of 5 nm and can simultaneously measure mechanical and electrical properties together with optical near-field signals. PF-SNOM is expected to facilitate three-dimensional nanoscale near-field characterizations and correlative in situ investigations on light-induced mechanical and electrical effects.

## Abstract (web summary)

Scanning near-field optical microscopy (SNOM) offers nanometer-scale spatial resolution, but generally does not retain tomographic information. Here, Wang et al. develop peak-force SNOM to section scattered fields and improve imaging resolution.

## Introduction

Scattering-type scanning near-field optical microscopy (s-SNOM) provides access to a variety of nanoscale phenomena that cannot be spectroscopically studied in situ by far-field spectroscopy due to the optical diffraction limit^1–3^. s-SNOM has been an essential tool for studying graphene plasmons^4–8^, surface phonon polaritons^9–13^, phase transitions in correlative electron materials^12,14–16^, compositions in heterogeneous materials^12,17–19^, and chemical reactions^20,21^. In s-SNOM, elastically scattered light from a sharp metallic tip operated in an atomic force microscope (AFM) over sample surface is measured by an optical detector^22,23^. Near-field interactions between the tip and sample modify the polarizability of tip, thus affecting the elastic scattering of light. However, elastic scattering does not change the wavelength of scattered light, so the reflected or scattered photons from other parts of the AFM cantilever or the sample surface outside the tip region are also registered by the same optical detector to create background signals, which are known as the far-field background. To differentiate near-field signals of tip−sample interactions from far-field background, the conventional approach is to oscillate the tip at the mechanical resonance of AFM cantilever in tapping mode and perform lock-in demodulation or Fourier analysis on scattered light at a non-fundamental harmonic of the tip oscillation frequency^22^.

Despite wide applications and successes, tapping mode s-SNOM with lock-in detection has limitations. First, conventional s-SNOM does not provide direct information on the range of tip−sample near-field interactions, as the s-SNOM signals coming out of lock-in demodulation are discrete values. Consequently, the distance dependence of tip−sample near-field interaction is convoluted in the signal generation mechanism and lost (see Supplementary Fig. 1 for more details). Second, s-SNOM signals from different demodulation orders could exhibit different signal shapes^24^ and result in ambiguity in spatial patterns (Supplementary Figs. 2−3). Moreover, the tapping mode operation of s-SNOM cannot simultaneously perform with other AFM modalities that require a firm tip−sample contact, such as measurement of mechanical properties and electrical conductivity. Simultaneous and correlative measurement of near-field optical, mechanical, and electrical signals are not possible for tapping mode s-SNOM apparatus.

To overcome these limitations, we developed a new type of s-SNOM, the peak force scattering-type near-field optical microscopy (PF-SNOM), to avoid tapping mode operation and subsequent information loss in lock-in detections. This method combines peak force tapping (PFT) mode^25,26^ and time-gated detection of near-field scattering signals with a far-field background subtraction algorithm. PF-SNOM enables tomographic sectioning of tip−sample near-field interactions with explicit tip−sample distance, which can be extended for three-dimensional mapping of near-field responses, as well as simultaneously performing correlative near-field, mechanical, and electrical measurements with high spatial resolution well below the diffraction limit.

## Results

### Demonstration of the principle of PF-SNOM

**Fig. 1:**
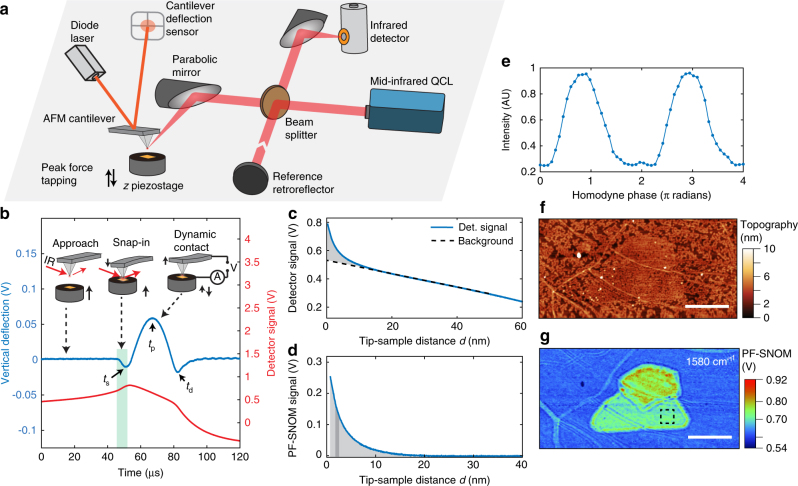
PF-SNOM and its imaging on graphene. **a** The peak force scattering-type scanning near-field optical microscopy (PF-SNOM) apparatus consists of a typical scattering-type scanning near-field optical microscopy (s-SNOM) setup coupled with an atomic force microscope (AFM) operated in the peak force tapping (PFT) mode. **b** Simultaneously recorded cantilever vertical deflection signal (blue curve) and infrared detector signal (red curve), revealing the change of light scattering signal during a PFT cycle. The green bar marks the snap-in contact motion concurrent with an increase of light scattering. The sample is a gold substrate. Time of the snap-in contact, maximal peak force and tip detachment are marked as *t*_s_, *t*_p_, and *t*_d_, respectively. The corresponding tip−sample configurations during three phases of a PFT cycle are shown, from the left to the right: the tip approaching sample, the snap-in contact and the dynamic tip−sample contact that allows other AFM modalities, such as the electrical measurement (as exemplified here by an applied voltage between tip and sample). **c** Relationship between the infrared detector signal and tip−sample distance *d* that is derived from two waveforms in **b**. Near-field enhancement at short *d* and a linear far-field background (dashed line) are observed. **d** Pure near-field signal with explicit distance dependence obtained by subtraction of the fitted linear background in **c**. Near-field response is read out from the integral of signal intensity under the curve at a specific tip−sample distance d (dark gray bar). **e** Homodyne phase dependence of PF-SNOM signals. **f** Topography of a region of chemical vapor deposition (CVD)-grown graphene on SiO_2_. The scale bar is 1 μm. **g** PF-SNOM image of the graphene region at 1580 cm^−1^ infrared frequency sectioned at *d* = 1 nm. The scale bar is 1 μm. The image reveals the distributions of plasmons, presence of multilayer graphene (large green and red areas), and boundaries of monolayer graphene (ribbon-like green areas). Each pixel in **g** is averaged from 150 PFT cycles

Figure 1a illustrates the PF-SNOM apparatus with a frequency tunable laser source, a stage-scan AFM, and an infrared detector and an interferometer. PF-SNOM uses the PFT mode as feedback mechanism. During operation, the cantilever is held stationary, and the AFM sample stage oscillates vertically with a large amplitude (for example, 300 nm) at a low frequency of several kilohertz (defined as the PFT frequency) by a piezo. The maximal cantilever deflection (peak force) is controlled and maintained as a set point under a negative feedback loop for each oscillation cycle^26^. Figure 1b displays simultaneously recorded cantilever vertical deflections (blue curve) and infrared detector signals (red curve) of the scattered light from the tip on a gold substrate by PF-SNOM. As the tip approaches the sample surface, the attractive intermolecular forces cause the tip to jump into contact with the surface, a phenomenon known as snap-in contact^27^. The time of snap-in contact *t*_s_ is well-defined and measured from shape of waveform of the cantilever deflection signal (blue curve in Fig. 1b). Then, a plot of scattering signal *S* versus tip−sample distance *d* is derived (as shown in Fig. 1c) by defining *d* to be zero at the snap-in contact point. A detailed algorithm of derivation procedure is described in the Methods section.

A clear increase of scattering signal raised by the short-range near-field interaction is observed in Fig. 1c, together with a linearly fitted long-range far-field background (dashed line). Because the PFT mode allows large amplitude oscillations of the tip−sample distance without losing feedback stability, the far-field background can be accurately fitted within a region where the tip is far away from the sample. Subtracting the linear background from the scattering signal provides the pure near-field response as a function of *d* (Fig. 1d). Alternatively, a direct background removal procedure in the time-domain is also feasible and computationally efficient (described in the Methods section). As a result, near-field signal can be sectioned from the near-field signal versus *d* curve at a defined tip−sample distance *d* (dark gray bar in Fig. 1d). By altering the location of extraction, corresponding near-field signals at different values of *d* can be obtained to capture the explicit vertical near-field response between the tip and sample.

Like conventional s-SNOM, PF-SNOM signal can be interferometrically detected with a homodyne reference field, which has been generally used to enhance the near-field signal and suppress background scattering in tapping mode s-SNOM. Figure 1e displays the PF-SNOM signal dependence at different homodyne phases by adjusting the position of reference retroreflector. As in tapping mode s-SNOM, the phase-sensitive PF-SNOM can also be used to compute amplitude and phase of the near-field signal, from which the absorption and reflection of sample may be extracted^22,28–30^. Note that PF-SNOM is also compatible with the newly developed synthetic near-field holography technique^31^ (Supplementary Fig. 4).

As a scattering-type near-field optical technique, PF-SNOM inherits the ability to study plasmon-polariton responses in two-dimensional (2D) materials by launching plasmon-polaritons and probing their near fields^4–6^. Figure 1f–g displays PF-SNOM measurements on a chemical vapor deposition (CVD)-grown graphene at an infrared frequency of 1580 cm^−1^ with a signal extracting tip−sample distance *d* of 1 nm. We can see clear differences in the near-field responses among monolayer, multilayer (possibly bilayer), and grain boundaries on the graphene^6^. The PF-SNOM signal reveals the plasmonic response of graphene and is in good agreement with conventional s-SNOM results (Supplementary Fig. 5).

The signal-to-noise ratio (SNR) calculated from a flat area on the multilayer graphene (black dashed box in Fig. 1g) is found to be 49, which is comparable to that of near-field signal from third harmonic demodulation of s-SNOM after normalization by using total acquisition time (see Supplementary Fig. 5 for the calculation), despite that the PF-SNOM operates at a much slower PFT frequency (2 kHz) than the tapping frequency (~ 220 kHz) in conventional s-SNOM. If one just considers the near-field signal per oscillation cycle, then the signal quality from PF-SNOM is considerably better than that in tapping mode s-SNOM to produce a comparable S/N ratio. This is because larger near-field signals are contained in PF-SNOM signal, given that extracted signal from PF-SNOM is proportional to magnitude of near-field signal itself, rather than the rapid change of near-field signal as the case in tapping mode s-SNOM via lock-in demodulation (as described in Supplementary Fig. 1).

### Tomographic near-field imaging on polaritonic materials

**Fig. 2:**
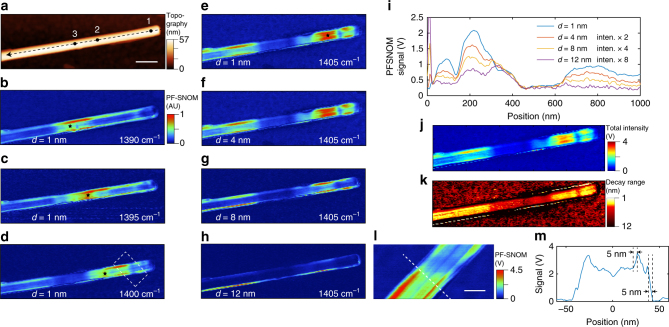
PF-SNOM on phonon polaritons of a boron nitride nanotube. **a** Topography of a boron nitride nanotube (BNNT). The scale bar is 200 nm and is the same for **a−h** and **j−k**. **b−e** Normalized peak force scattering-type scanning near-field optical microscopy (PF-SNOM) images of the BNNT sectioned at tip−sample distance *d* = 1 nm at 1390, 1395, 1400, and 1405 cm^−1^ infrared frequencies respectively. The color bars for **b−h** are all the same. Maxima of near-field signals on the top surface of BNNT (indicated by black stars) shift toward the right terminal as infrared frequency increases. **f−h** PF-SNOM images of the BNNT at 1405 cm^−1^ at *d* = 4, 8 and 12 nm. **i** Extracted profiles of PF-SNOM responses from the top surface of BNNT at 1405 cm^−1^ in **e**−**h** along the black dashed vector in **a**. For the convenience of comparison, profiles from *d* = 4, 8, and 12 nm are magnified by factors of 2, 4, and 8 respectively. **j** Map of near-field amplitude *A*_NF_ of the BNNT calculated from **e−h**. **k** 1/e decay range of the near-field amplitude of BNNT calculated from **e−h**. **l** Zoomed-in PF-SNOM image of the BNNT, the same region is indicated by a white dashed box in **d**. The scale bar is 50 nm. All PF-SNOM images are acquired using an averaged signal of 50 PFT cycles per pixel. **m** PF-SNOM signal profile across the BNNT as indicated by the white dashed line in **l**, a spatial resolution of 5 nm is observed

Surface phonon polaritons (PhPs) are surface electromagnetic modes formed by collective oscillations of optical phonons and the electric field bound to the surface. Polar materials such as silicon carbide (SiC) and boron nitride (BN) are known to support surface PhPs^10^. PF-SNOM is capable of probing PhPs on a sample surface in both lateral and vertical directions. Figure 2 shows the measurement of a boron nitride nanotube (BNNT) using PF-SNOM (conventional s-SNOM images are shown in Supplementary Fig. 6). Topography of the BNNT is displayed in Fig. 2a. Figure 2b–e shows PF-SNOM images at infrared frequencies from 1390 to 1405 cm^−1^ with an extracting tip−sample distance *d* *=* 1 nm. In these figures, the presence of PhPs is revealed, as the maxima locations of near-field signal (marked by black stars in Fig. 2b–e) move towards the nanotube terminal as infrared frequency increases. This phenomenon is due to the fact that spatial contrast from near-field scattering is generated by the reflected tip-launched PhP waves from the nanotube terminal^10^: as polaritonic wavelength decreases with increasing infrared frequency, the constructive interference positions (signal maxima) shift toward the tube terminal.

Figure 2e–h shows tomographic PF-SNOM images extracted at *d* *=* 1, 4, 8, 12 nm at the infrared frequency of 1405 cm^−1^. To better reveal the spatial distribution of the near-field responses as a function of tip−sample distance *d*, PF-SNOM signal profiles along the long axis of BNNT (indicated by the dashed vector in Fig. 2a) are shown in Fig. 2i. As *d* decreases from 12 to 1 nm, the near-field profiles along the top surface of nanotube show not only overall larger near-field signal as expected, but also more fraction of near-field intensities closer to the right terminal of the nanotube (Fig. 2i), which indicates stronger tip−sample interactions are available at the terminal of BNNT when tip gets closer to the surface. By fitting the vertical decay behavior of near-field responses with an empirical exponential decay function $S( d ) = A_{{\mathrm{NF}}}{\mathrm{e}}^{ - d/b}$, where *S*(*d*) is the near-field response that depends on tip−sample distance *d*, and $A_{{\mathrm{NF}}}$ and *b* are fitting coefficients representing total amplitude of near-field response and vertical decay range, we can obtain two new representations of near-field signals based on the total near-field amplitude $A_{{\mathrm{NF}}}$ (Fig. 2j) and the tip−sample characteristic $1/{\mathrm{e}}$ decay range *b* (Fig. 2k). Figure 2k reveals that the near-field interactive range between the tip and the polaritonic BNNT is spatially dependent on near-field probing positions. The observation may indicate the presence of both surface PhPs and volume hyperbolic PhPs^9^ that have different near-field interaction ranges along spatial locations of BNNT. The direct acquisition of tip−sample interaction range is a unique advantage of PF-SNOM method, while tapping mode s-SNOM with individual lock-in demodulation lacks such capability, if no further reconstruction treatment of near-field response is involved^32^.

**Fig. 3:**
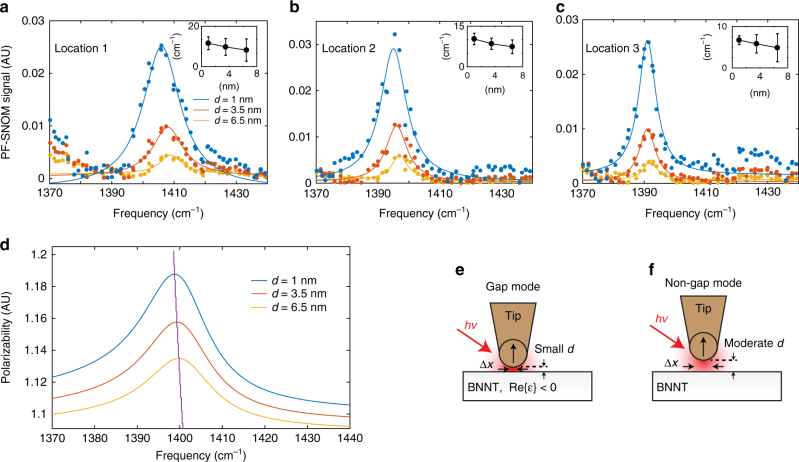
Resonant peak positions and spectral widths of a boron nitride nanotube. **a**−**c** Spectra from locations 1, 2, and 3 on the boron nitride nanotube (BNNT) marked in Fig. 2(a) at three values of *d* of 1 nm (blue), 3.5 nm (red), and 6.5 nm (yellow) are shown as dots. Lorentzian fittings on the central peak regions are shown as solid curves of corresponding colors. The insets show the transition of full-width at half-maximum (FWHM) linewidths of the fitted resonant peak *ω*_p_ at three different values of *d*. As *d* decreases, an increase of the linewidth of *ω*_p_ is observed. **d** Simulated spectra based on the image dipole model at *d* = 1, 3.5, and 6.5 nm with the dielectric function of boron nitride (BN) from Geick et al.^33^. A shift of *ω*_p_ at different values of *d* is observed and shown by the purple line. **e** Schematic illustration of excitation of the gap mode between tip and sample when *d* is very small. Laterally localized electric field with high confinement is generated within the tip−sample gap with the real part of the dielectric function of BN below zero. The scale of field enhancement is much smaller than the radius of metallic tip. **f** Schematic illustration of the electric field of tip when *d* is moderately large. The gap mode enhancement is absent, and the field enhancement is determined by the radius of metallic tip

Spatial resolution of PF-SNOM is estimated from the edge of the BNNT. Figure 2l displays a zoomed-in region of the white dashed box in Fig. 2d, with *d* of 1 nm. Signal profile of the PF-SNOM response along the white dashed line in Fig. 2l is shown in Fig. 2m, where a spatial resolution of 5 nm is obtained. In comparison, the metallic tip used in this study is estimated to have a radius of 30 nm, and the spatial resolution of tapping mode s-SNOM at third harmonic is found to be 13 nm (s-SNOM results for second to fifth harmonics and extracted profiles for spatial resolution estimation are included in Supplementary Figs. 6−7). The great improvement of the spatial resolution of PF-SNOM is due to the gap-mode enhancement at the short tip−sample distance of 1 nm formed by highly non-uniform spatial distribution of light field, which is tightly bound in lateral dimension in a range much less than radius of tip, as illustrated in Fig. 3e.

The spectral response of BNNT also exhibits dependence on the tip−sample distance *d*. Figure 3a−c shows measured scattering spectra from three locations on the BNNT (marked as 1, 2, 3 in Fig. 2a) by PF-SNOM at three different values of *d* at 1, 3.5, and 6.5 nm with infrared frequencies from 1370 cm^−1^ to 1440 cm^−1^. Fitting parameters for the PF-SNOM spectra from three locations and three tip−sample distances are tabulated in Table 1, in which one can find that as the probing locations get further away from the terminal of nanotube (from location 1 to 3), the central resonant frequency *ω*_p_ of the PhPs redshifts and its peak linewidth narrows (in terms of full-width at half-maximum (FWHM)). This is also a result from the interference between tip-launched PhPs and terminal-reflected PhPs as above described. PhPs stimulated by lower infrared frequencies have longer polariton wavelengths and lower losses. Such PhPs with lower frequencies travel further and thus are more prominent at locations further away from the terminal. On the other hand, as the lower loss of polaritons corresponds to a longer lifetime, the linewidth of the resonant peak *ω*_p_ is also consequently narrower. These two features should also be detected by conventional tapping mode s-SNOM but is not further discussed here.

**Table 1:** Resonant peak *ω*_p_ and its linewidth as functions of tip−sample distance *d* and probing locations on the boron nitride nanotube (BNNT), fitted and summarized from Fig. 3a–c

Location	Location 1		Location 2		Location 3	
	FWHM (cm^−1^)	*ω*_p_ (cm^−1^)	FWHM (cm^−1^)	*ω*_p_ (cm^−1^)	FWHM (cm^−1^)	*ω*_p_ (cm^−1^)
*d* (nm)						
1	13.7 ± 2.4	1406 ± 1	10.3 ± 2.1	1395 ± 1	7.3 ± 0.8	1391 $\pm$ 1
3.5	12.3 ± 3.1	1408 ± 1	8.5 ± 2.0	1396 ± 1	6.6 ± 1.8	1391 $\pm$ 1
6.5	11.1 ± 4.1	1409 ± 1	7.4 ± 2.5	1397 ± 1	5.8 ± 2.7	1392 $\pm$ 1

There are two special features involving the tip−sample distance in Table 1 that can only be revealed by PF-SNOM. First, at the same location on the BNNT, the resonant frequency *ω*_p_ of PhPs redshifts by a small amount when the tip−sample distance *d* decreases. This shift can be qualitatively interpreted by the image dipole model based on the dielectric function of boron nitride^33^. Figure 3d shows simulated relationship between maxima of polaritonic resonance spectra and tip−sample distance *d* according to the image dipole model with a 30-nm radius Au-coated tip^22,34^. A small shift of 1 cm^−1^ of *ω*_p_ is observed in Fig. 3d with tip−sample distance *d* decreased from 6.5 to 1 nm. Because the spatial frequency of PhPs depends on the excitation infrared frequency, the shift of *ω*_p_ suggests that spatial patterns of PhPs should change at different *d* of measurement in high dispersion region of PhPs. We have observed such an effect with PF-SNOM (Supplementary Fig. 8).

The second feature from Table 1 is that at the same probing location, the linewidth of *ω*_p_ broadens when the tip−sample distance *d* decreases, indicating that the lifetime of the BNNT PhPs decreases as the tip gets closer to the surface. The reduction of the lifetime is possibly due to more relaxation channels formed by the presence of highly confined gap mode, which will considerably increase optical density of states between the tip apex and the surface of the BNNT. Figure 3e–f schematically illustrates the formation of the gap mode depends on the tip−sample distance: at the PhP-active frequency and a small tip−sample distance of less than several nanometers, the real part of the dielectric function of BN is negative and a strong gap mode enhancement is formed between metallic tip and the surface of BN. Energy in the PhPs is likely to partition in the increased optical density of states in the gap mode and couple to additional relaxation channels, such as radiation via the metallic tip acting as a nano-antenna. Consequently, the relaxation of PhPs is favored and the linewidth of the resonances broadens, despite that the strong field enhancement of gap mode will excite more PhPs. This enhanced relaxation effect is conceptually similar to the Purcell effect of fluorophore coupled to a resonant cavity^35^ and tip-enhanced relaxation^36,37^. Note that the linewidth broadening is not seen with the image dipole model simulation in Fig. 3d, because the image dipole model does not consider additional relaxation channels at short tip−sample distance. We will later discuss a modified image dipole model that accounts for tip damping that reproduces the linewidth broadening effect.

**Fig. 4:**
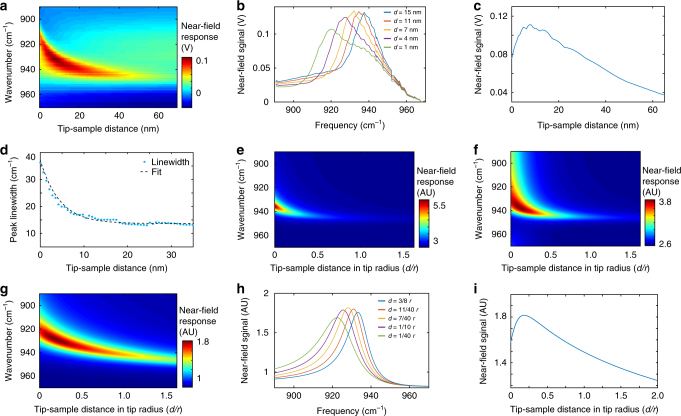
Distance dependence of near-field spectra of SiC revealed by PF-SNOM and modified models. **a** Experimentally collected two-dimensional near-field response of SiC that depends on the tip−sample distance *d* and infrared frequencies. Redshift and broadening of the resonant peak are observed as *d* decreases. The peak-force scattering-type scanning near-field optical microscopy (PF-SNOM) data are acquired using an averaged signal of 400 peak force tapping (PFT) cycles per pixel. **b** Extracted PF-SNOM spectra of SiC at *d* = 1, 4, 7, 11, and 15 nm. **c** Plot of peak intensities of the near-field spectra versus *d*. A reduction of peak intensity is observed for *d* below 8 nm. **d** The relationship between linewidth of the resonance peak and *d* from experiment measurement (blue dots). A clear increase of the spectral linewidth is observed as *d* decreases. A fit by an exponential function with an offset is shown as the dashed curve. **e** Simulated relationship between near-field response, *d* and infrared frequency using the image dipole model^22, 34^ and dielectric function of SiC^39^, which exhibits a clear redshift of the resonance peak as *d* decreases. However, the feature of linewidth broadening is not captured by the image dipole simulation. **f** Simulation result with a modified image dipole model, with *a*′ set as 0.5 and *b*′ set as 20% of the tip radius. **g** Simulated result with the modified finite dipole model with *a*′ set as 0.05 and *b*′ set as 10% of the tip radius. **h** Near-field spectra of SiC at different tip−sample distances extracted from the simulation in (**g**), the shift and broadening of SiC spectra are reproduced, showing the same trend with experimental results (**b**). **i** Near-field resonant peak intensity as a function of *d* extracted from the simulation by modified finite dipole model in **g**, which reproduces the damping feature at short *d* observed in the experiment extraction in **c**

SiC is another material that supports PhPs in the mid-infrared range^38,39^. Similar to the BNNT, we study SiC with PF-SNOM to reveal spectral features that depend on tip−sample distance which are otherwise difficult to access for conventional tapping mode s-SNOM. Figure 4a presents experimentally collected two-dimensional infrared scattering signals of SiC versus tip−sample distance *d* (as the horizontal axis) and infrared frequency from 900 to 960 cm^−1^ (as the vertical axis). The near-field spectra of SiC from PF-SNOM are shown in Fig. 4b for different values of *d* ranging from 1 to 15 nm. An obvious redshift of resonant peak is observed. This redshift qualitatively agrees with the phenomenon of nanomechanical tuning observed by Taubner et al. with tapping mode s-SNOM^39^. Peak intensities of near-field scattering spectra versus tip−sample distance *d* are plotted in Fig. 4c. As *d* decreases, the near-field scattering signal first increases to a maximum at *d* = 8 nm, and then decreases within a short range. The decrease of the scattering signal suggests increased damping at short tip−sample distances. Figure 4d shows peak linewidth $\Delta \omega _d$ (in terms of FWHM) versus *d* in blue dots. A clear increase of linewidth is observed within *d* < 10 nm. An exponential fit with an offset ($\Delta \omega _d = \Delta \omega _{d = 0}{\mathrm {e}}^{ - d/b_{\mathrm{t}}} + \Delta \omega _{d = \infty }$, *d* is the tip−sample distance, $\Delta \omega _{d = 0}$, $b_{\mathrm{t}}$, and $\Delta \omega _{d = \infty }$ are fitting parameters) is performed and shown as a dashed curve in Fig. 4d. The offset $\Delta \omega _{d = \infty }$, which represents the linewidth of peak at a large tip−sample distance where it plateaus, is found to be 14 cm^−1^. The 1/e decay range of the linewidth (the fitting coefficient $b_{\mathrm{t}}$) is found to be 9 nm. The non-monotonic trend and the increase of the linewidth are not interpretable by either image dipole model (Fig. 4e) or finite dipole model (Supplementary Fig. 9)^22^. The simulated responses from the image dipole model only show a monotonic increase of the scattering signal as *d* decreases, but the linewidth remains the same rather than exhibiting significant increase as the experimental observation.

Measurements from both BNNT and SiC exhibit an increase of peak linewidth as the tip−sample distance decreases, which is not predicted by the image dipole model or the finite dipole model^40,41^. To conceptually capture the increased damping at small *d* in tip−sample near-field interaction, we modify the image dipole model by including a distance-dependent damping term to the polarizability of tip. This damping term corresponds to conversion of energy from the tip−sample interaction to other forms of energy that dissipate to surroundings, such as radiative emission via the tip acting as an antenna. In the well-known image dipole model^22,34^, the effective polarizability $\alpha _{{\mathrm{eff}}}$ is given by Eq. (1):

1 $$\alpha _{{\mathrm{eff}}} = \alpha \left(1 - \frac{{\alpha \beta }}{{16{\mathrm{\pi }}(r + d)^3}}\right)^{ - 1},$$

where $\alpha = 4{\mathrm{\pi }}r^3(\varepsilon _{\mathrm{t}} - 1)/(\varepsilon _{\mathrm{t}} + 2)$ is the tip polarizability and $\beta = (\varepsilon _{\mathrm{s}} - 1)/(\varepsilon _{\mathrm{s}} + 1)$ is the sample polarizability, with tip radius $r$, tip−sample distance *d*, dielectric function of the tip $\varepsilon _{\mathrm{t}}$ and dielectric function of the sample $\varepsilon _{\mathrm{s}}$. The radiative emission of energy through the metallic tip as a nano-antenna leads to dissipation of energy that should be dependent on *d*. The shorter the distance, the stronger the damping/dissipation. Thus, we introduce a modification term to the tip polarizability $\alpha$ to account for dissipative imaginary part at short tip−sample distance. For simplicity, we multiply $\alpha$ with a *d*-dependent factor ${\mathrm{e}}^{i\varphi (d)}$ in Eq. (1) to obtain the modified polarizability of the tip $\alpha _{{\mathrm{mod}}}$, which is shown in Eq. (2):

2 $$\alpha _{{\mathrm{mod}}}\left( d \right) = \alpha {\mathrm {e}}^{i\varphi (d)} = 4{\mathrm{\pi }}r^3\frac{{\varepsilon _{\mathrm{t}} - 1}}{{\varepsilon _{\mathrm{t}} + 2}}{\mathrm{e}}^{i\varphi (d)}.$$

The phase factor $\varphi (d)$ represents the rotation of polarizability of tip into the imaginary (dissipative) part and should be zero for a large value of *d* and have finite values as *d* decreases. Note that when $\varphi (d)$ is small, the modified $\alpha _{{\mathrm{mod}}}$ can also be expressed as $\alpha _{{\mathrm{mod}}}( d ) \approx \alpha + i\alpha \varphi ( d )$. Because we do not know the exact form of $\varphi ( d )$ a priori, for simplicity, we use an exponential form of $\varphi ( d ) = a\prime {\mathrm{e}}^{ - d/b\prime }$to describe the *d* dependence with amplitude factor $a\prime$ and characteristic 1/e decay range $b\prime$. A large value of $a\prime$ means strong dissipation through the tip. This exponential form satisfies the constraint that it decays to zero at large values of *d*. We then replace $\alpha$ in Eq. (1) with the modified $\alpha _{{\mathrm{mod}}}( d )$ to calculate the *d*-dependent $\alpha _{{\mathrm{eff}}}(d)$ in Eq. (3):

3 $$\alpha _{{\mathrm{eff}}}(d) = \alpha _{{\mathrm{mod}}}( d )\left(1 - \frac{{\alpha _{{\mathrm{mod}}}( d)\beta }}{{16{\mathrm{\pi }}(r + d)^3}}\right)^{ - 1}.$$

Equation (3) is then used to calculate SiC scattering spectra versus tip−sample distance *d*. The resulting two-dimensional scattering response is shown in Fig. 4f. The simulation with modified image dipole model successfully reproduces three key features observed in the experiment: (1) The red shift of polaritonic resonance peak; (2) The broadening of linewidth as *d* decreases; (3) The reduction of maxima (peak intensities) of the polaritonic resonance as *d* decreases in a short range (similar to Fig. 4c, i).

Note that the image dipole model^22,34^ is known to predict near-field interactions with range shorter than that obtained from experiment^32,40^. This bias also manifests in our simulation comparing to the experimental observations. The finite dipole model^40,41^ is another popular model for tip−sample near-field interactions with improved accuracy albeit at a cost of more complexity. We then extend a similar treatment to the tip polarizability in the finite dipole model. Details of the modification are included in Methods section. Figure 4g shows the two-dimensional scattering response by the modified finite dipole model, aforementioned three key features are also reproduced here. The finite dipole model renders near-field interactions in a larger tip−sample distance range compared with the image dipole model and is closer to the experimental results. To make things clear, Fig. 4h shows the simulated near-field spectra sectioned from Fig. 4g at five different tip−sample distances, in which both red shift of the resonance peak and linewidth broadening can be observed as *d* decreases. Figure 4i shows the relationship between modeled maxima (peak intensities) of the near-field spectra and tip−sample distance *d*, where a signature of damping is reproduced within short tip−sample distance, which is consistent with the experimental observation.

### Correlative mechanical and electrical properties mapping

**Fig. 5:**
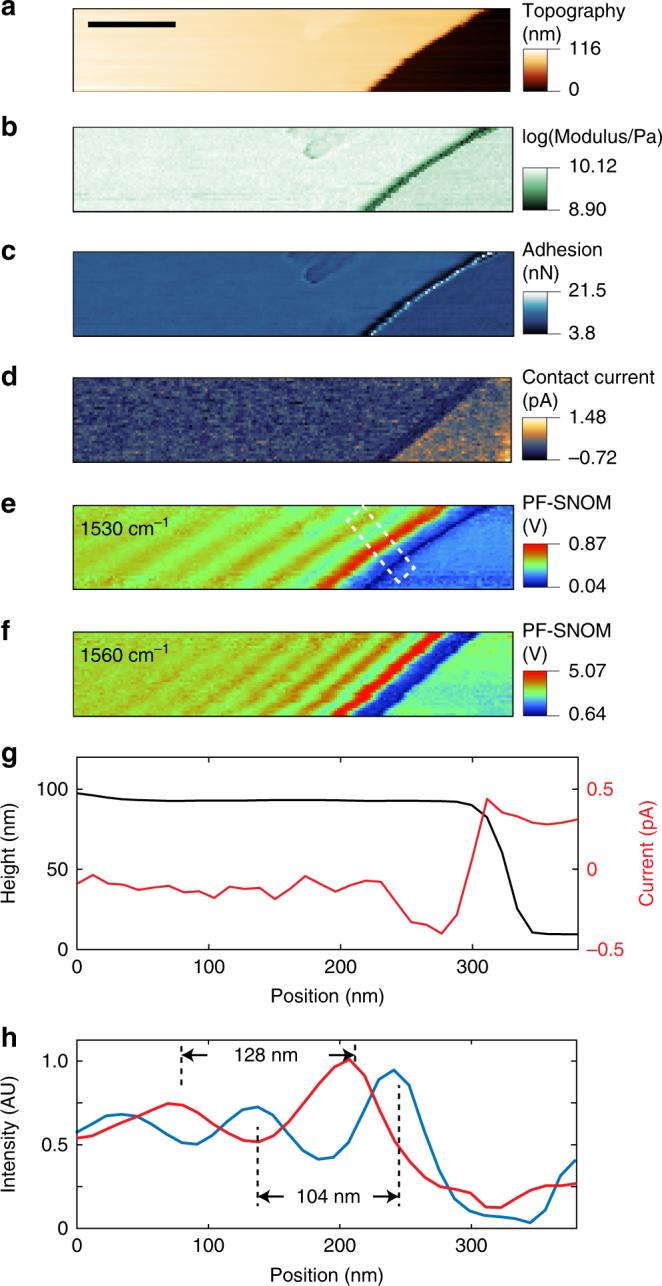
Multimodal capability of PF-SNOM with correlative mechanical, electrical, and optical near-field measurements. **a** Topography of a multi-layer hyperbolic boron nitride (h-BN) on an Si substrate. The scale bar is 400 nm and is the same for **b−f**. **b** Logarithmic modulus map of h-BN and Si surface. **c** Map of adhesion between the tip and the sample surface. **d** Electrical contact current between the tip and the sample surface measured in the peak force tapping (PFT) operation. **e−f** Peak force scattering-type scanning near-field optical microscopy (PF-SNOM) images at 1530 and 1560 cm^−1^ infrared frequencies. Phonon polaritons of h-BN are revealed. Every pixel in the PF-SNOM images is an averaged signal of 150 PFT cycles. **g** Profiles of topography (black) overlaid with electrical current (red), the profile is averaged across the interface between h-BN and Si from an area shown as a white dashed box in **e**. **h** Average profiles of PF-SNOM responses of 1530 cm^−1^ (red) and 1560 cm^−1^ (blue) from the white dashed box in **e**. The spatial periods of polaritonic patterns of h-BN are found to be 128 nm for 1530 cm^−1^ and 104 nm for 1560 cm^−1^, which correspond to the half wavelength of phonon polariton wave in h-BN

s-SNOM requires tapping mode operation, which is not compatible with conductive AFM or mechanical measurements that require a firm tip−sample contact. Now with PF-SNOM, all three AFM modalities including optical near-field characterization can be simultaneously performed thanks to the dynamic contact of PFT mode. Figure 5 shows such multimodal measurement of a sheet of hexagonal boron nitride (h-BN) with an 83-nm thickness on a Si substrate. Topography, modulus, adhesion, contact current, and PF-SNOM images at 1530 and 1560 cm^−1^ are presented in Fig. 5a–f, respectively. The multimodal measurement reveals that the h-BN is more rigid than the Si substrate, exhibits higher tip−sample adhesions than Si, is less conductive than Si, and supports PhPs in mid-infrared. Signal profiles of contact current and polaritonic response are plotted in Fig. 5g–h, respectively. From 1530 to 1560 cm^−1^, the spatial period of PhP patterns reduces from 128 to 104 nm, which is consistent with the dispersion relation of hyperbolic polaritons of h-BN in literature^9^. This measurement demonstrates the multimodal spectroscopic, electrical, and mechanical characterization capabilities of PF-SNOM, which provide PF-SNOM versality to investigate correlative light−matter interactive phenomena. For example, we have found that apparent mechanical modulus of a BNNT may correlate with light-induced PhPs and show different periodical spatial patterns as a function of the frequency of incident light (see Supplementary Fig. 10). Such correlative phenomena are conveniently accessed by PF-SNOM, and worth more investigations in future studies.

## Discussion

The main advantage of PF-SNOM over conventional tapping mode s-SNOM is its ability to directly acquire near-field scattering signals with the vertical tip−sample distance dependence. This ability allows collections of tomographic near-field images, which can reveal more subtlety of tip−sample interactions than s-SNOM. Linewidth broadening and resonant frequency shift of PhP resonances in BNNT and SiC are observed by PF-SNOM within a tip−sample distance range of less than 10 nm, a range that is very difficult for tapping mode s-SNOM to distinguish due to necessary oscillations of the tip and subsequent convoluted signal generations. Because direct and quantitative elastic scattering signals can be obtained by PF-SNOM, we are able to study features of tip-induced relaxation of PhPs and modify the image dipole model and the finite dipole model to account for the tip-induced damping/dissipation effect.

As PF-SNOM signal is directly proportional to the near-field signal, numerical modeling of PF-SNOM signals requires much less computational complexity than tapping mode s-SNOM, where additional steps are needed to account for tip oscillations and lock-in demodulations to reproduce the s-SNOM signal^7,41,42^. Although the approach of reconstruction s-SNOM with Fourier synthesis has been used with tapping mode s-SNOM to vertically reconstruct tip−sample near-field responses^32^, the bandwidth of reconstruction s-SNOM is limited by the number of harmonic demodulations in the Fourier synthesis and the oscillation amplitude of tip. For example, even using a total of 18 harmonic demodulations, vertical resolution in the reconstruction s-SNOM is only 8.3 nm for a 150-nm tip oscillation amplitude. A vertical resolution of 8.3 nm is unable to capture the features of near-field interactions in a short range below 10 nm as presented in this work. Let alone to simultaneously register 18 harmonics, expensive high-end multi-channel lock-in amplifiers are necessary. In comparison, vertical resolution of PF-SNOM is related to bandwidth of the infrared detector, PFT amplitude, and PFT frequency, all of which can be easily tuned or satisfied to enhance vertical resolution. In our PF-SNOM apparatus, the vertical resolution is estimated to be 0.12 nm; thus PF-SNOM is more accurate for resolving near fields in the vertical direction than existing tapping mode s-SNOM technique^32^. Large numbers of tomographic images within a large range of tip−sample distance can be directly obtained by PF-SNOM. This advantage can be used to reveal three-dimensional near-field distribution of plasmonic nano-antennas^43^. Furthermore, because the spatial resolution is determined by the lateral confinement of field enhancement^44^, PF-SNOM with high vertical precision allows achieving the tightest field confinement in the gap mode by acquisition at short tip−sample distances, and provides superior lateral spatial resolution of 5 nm over that of 10−20 nm of tapping mode s-SNOM, where the size of the field confinement changes as the tip is vertically oscillating, leading to a compromised lateral field confinement.

Tomographic near-field images of BNNT and spectra of SiC by PF-SNOM have implications for scattering-type near-field optical microscopy. s-SNOM is widely used in the characterization of near-field responses of polaritonic materials. It has been rarely a concern that the AFM tip can change near-field behaviors of samples. In PF-SNOM where near-field responses at short tip−sample distance are directly extracted, changes of the spectral behaviors of PhPs are clearly observed. Our observation as well as the modified near-field interaction models suggest that the metallic tip is not just a scattering object for probing the near field, but also acting as a damper at short tip−sample distance. This means the spatial patterns obtained by scattering-type near-field optical microscopy in general should be treated with caution, as the measuring tip can affect the near-field responses that are being measured. The measurement made by PF-SNOM, because of the ability to acquire responses at different tip−sample distances, should be particularly useful in deciphering actual near-field responses.

The ability to acquire vertical near-field responses as well as the compatibility with simultaneous mechanical and electrical measurement of PF-SNOM stem from real-time measurement of near-field scattering signals, which is enabled by a well-defined tip−sample contact in the PFT mode. As the cantilever of AFM tip is held stationary, except being pushed upward during the dynamic contact, the kinetic energy stored in cantilever is almost zero. Consequently, the response of cantilever to intermolecular force between tip and sample is highly sensitive, thus giving out a well-defined snap-in contact for the zero tip−sample distance reference point. In comparison, the cantilever in tapping mode AFM is externally driven to oscillate. The intermolecular force between tip and sample leads to a considerable shift of the oscillation phase of the cantilever from the external driving oscillation. While the phase shift in tapping mode AFM is informational and used as phase imaging mode^45^, the large phase shift creates a problem to determine the timing of momentary tip−sample contact for possible utilization for other AFM modalities. The shifting oscillatory phase in tapping mode AFM also renders a time-gated detection scheme difficult.

In tapping mode AFM, the repulsive force, attractive force, and especially adhesive force between the tip and sample lead to nonlinear dynamic behaviors of the cantilever oscillations^46–48^, which will also manifest even in high-order lock-in demodulations via anharmonic far-field scattering. In contrast, the PF-SNOM directly measures the scattering signal from the approaching side of the PFT cycle, where the adhesion between the tip and sample after contact does not have any effect, thus avoiding possible mechanical distortions from sticky surfaces. In this regard, PF-SNOM is more adaptive to rough and sticky samples than tapping mode s-SNOM.

PF-SNOM is complementary to the existing peak force infrared (PFIR) microscopy that measures the laser-induced photo-thermal expansion in the PFT mode^49^. PF-SNOM optically detects the near-field scattering signal from the tip and sample that is determined by the tip−sample polarizability and propagating surface waves; whereas PFIR performs mechanical detection on the result of local optical absorption and subsequent dissipation of energy into heat. PF-SNOM inherits the advantages of s-SNOM in the measurement of rigid and polaritonic materials with spatial contrast in dielectric functions, in comparison to PFIR microscopy that is suitable for soft matters with large photo-thermal expansion coefficients.

PF-SNOM enables simultaneous mechanical, electrical and optical near-field characterization of samples. Three complimentary aspects of properties of the sample obtained in one measurement by PF-SNOM will be very useful in investigations of nanoscale behaviors of functional materials, such as the metal−insulator transitions of correlated electron materials^14,16,50–52^, and nanoscale optical mechanical structures and devices^53,54^. The versatile compatibility makes PF-SNOM a more complete scattering-type near-field optical microscopic platform for many applications.

## Methods

### PF-SNOM apparatus

An s-SNOM apparatus was built with an AFM (Multimode 8 AFM, Bruker Nano), an asymmetric Michelson interferometer with a mercury cadmium telluride infrared detector (KLD series, Kolmartech), and a frequency tunable quantum cascade laser (MIRcat, Daylight Photonics) according to literature^23^ and schematically shown in Fig. 1a. Gold-coated AFM probes (HQ:NSC15/CR-AU BS, Mikromasch) were used to enable near-field enhancement. The infrared beam was focused with a parabolic mirror (numerical aperture of 0.25) to the tip−sample region of AFM. Scattered light was collected by the same parabolic mirror to reach the infrared detector after interferometric amplification. The optical path of reference beam in the interferometer was adjusted to maximize signal. In PF-SNOM operation, the AFM was operated in the PFT mode at 4 kHz with the same tip with a peak force amplitude of 50 nm and peak force setpoint from 4 to 20 nN. Unlike in tapping mode operation, the cantilever of AFM probe was not externally driven, instead, it was the sample that was oscillated by a piezo vertically to the stationary probe. The peak-to-peak oscillation amplitude of the sample stage was 300 nm. The vertical deflection signal of cantilever was read from the position sensor of a quadrant photodiode of AFM and routed to a two-channel data acquisition card (PXI-5122, National Instruments, operated at 50 M samples per second). The same data acquisition card also simultaneously recorded voltage signal from the infrared detector. The snap-in contact of cantilever was identified from its vertical deflection signal and used as reference time point. The gated detection was made on the infrared scattering signal prior to the snap-in contact and processed to obtain PF-SNOM signal. The operational speed of PF-SNOM linearly scales with the PFT frequency, which is currently limited up to 4 kHz for the Bruker Multimode 8 AFM. Acquiring one PF-SNOM image of 256 × 256 pixels required about 30 min for the current implementation. In PF-SNOM imaging, an average signal of 50 or 150 cycles was used per pixel according to the signal strength. The PF-SNOM spectra were collected by sweeping frequencies of the tunable quantum cascade laser while keeping the PFT frequency at 2 kHz. Each point in PF-SNOM spectra was an average of signals from 400 PFT cycles.

The conventional s-SNOM measurements were conducted by operating the AFM in the tapping mode feedback at 220 kHz, which was the resonant frequency of cantilever. The peak-to-peak oscillation amplitude of cantilever was set to 30 nm. The voltage signal waveform from the infrared detector was demodulated with a lock-in amplifier (HF2Li, Zurich Instruments) at different harmonics of the tapping frequency. The demodulated signals from lock-in amplifier were used as conventional s-SNOM signals to form images in Supplementary Figs. 2−3, and 5−7.

### Procedure to obtain the dependence of scattering signal on *d*

A procedure was developed to convert the cantilever vertical deflection waveform $D(t)$ and the detector signal waveform of the scattered light $S(t)$, where $t$ is the time, into a function $S(d)$ where $d$ is the tip−sample distance (see Fig. 1c) for tomographic imaging. In PFT mode, the sample piezo-stage was vertically driven in a sinusoidal oscillation at the PFT frequency $f$. The vertical position of the piezo-stage $d_{\mathrm{z}}$ can be written as:

4 $$d_{\mathrm{z}}(t) = A{\mathrm{cos}}(2{\mathrm{\pi }}ft + \varphi ),$$

where $A$ is the peak force amplitude and $\varphi$ is the oscillation phase of the piezo-stage. The highest position of the piezo-stage also corresponds to the maximal cantilever vertical deflection (peak force set point) at the time $t_{\mathrm{p}}$. At $t_{\mathrm{p}}$, the phase of piezo-stage $\varphi$ is calculated from $2{\mathrm{\pi }}ft_{\mathrm{p}} + \varphi = 0$ for maximal $d_{\mathrm{z}}$ and then derived as $\varphi = - 2{\mathrm{\pi }}ft_{\mathrm{p}}$. Therefore, the vertical position of the piezo-stage $d_{\mathrm{z}}$ is:

5 $$d_{\mathrm{z}}(t) = A\cos \left( {2{\mathrm{\pi }}f(t - t_{\mathrm{p}})} \right).$$

In the experiment, the time $t_{\mathrm{p}}$ of maximal cantilever bending was identified from the cantilever vertical deflection signal waveform $D(t)$. With the knowledge of deflection sensitivity $V$ of the cantilever with a typical value of several tens of nm V^−1^, the tip−sample distance in time domain $d(t)$ is then derived as:

6 $$d(t ) = A - d_{\mathrm{z}}\left( t \right) + V \cdot D(t)$$

or,

7 $$d( t) = A - A\cos \left( {2{\mathrm{\pi }}}f(t - t_{\mathrm{p}}) \right) + V \cdot D(t)$$

Because the tip−sample distance was practically zero (we did not consider the role of indentation in PF-SNOM, as the signal was not extracted during indentation) after the snap-in contact time $t_{\mathrm{s}}$ and before the detachment time $t_{\mathrm{d}}$, we can define $d(t)$ into two regions, in a truncated sinusoidal shape given by:

8 $$d(t) = \left\{ {\begin{array}{*{20}{c}} {A - A\cos \left( {2{\mathrm{\pi }}}f(t - t_{\mathrm{p}}) \right) + V \cdot D(t),\;t\, < \, t_{\mathrm{s}}\;{\mathrm{and}}\;t > t_{\mathrm{d}}} \\ {0,\;t_{\mathrm{s}} \le t \le t_{\mathrm{d}}} \end{array}} \right.$$

All $t_{\mathrm{p}}$, $t_{\mathrm{s}}$, and $t_{\mathrm{d}}$ can be identified from the original $D(t)$ curve. In our current PF-SNOM operation, only the light scattering signal $S(t)$ before the snap-in contact time $t_{\mathrm{s}}$ was processed for each PFT cycle. Therefore, from the calculated $d(t)$ and the synchronously measured $S(t)$ for *t*<*t*_s_, the relationship between the scattering signal and the tip−sample distance $S(d)$ was derived.

### Procedure to remove far-field background

The far-field background was linearly fitted in the region of $S(d)$ where the tip−sample distance $d$ was large as shown in Fig. 1c. At a large tip−sample distance, the change of detector signal was from the change of far-field scattering from the cantilever shaft and the sample surface. As the wavelength of the infrared laser was about 6−12 µm in our studies, the change of position of sample stage by several tens of nanometers was relatively small, so the change of far-field scattering signal should be linear with respect to the tip−sample distance. The fitted linear far-field background was then extrapolated to the region of short tip−sample distance to be subtracted from the detector signal. The resulting difference (Fig. 1d) provides the relationship $S(d)$ between the near-field signal and the tip−sample distance that enables explicit access to the vertical near-field response.

### Fast algorithm to obtain PF-SNOM signal

An alternative, fast background removal algorithm was developed to directly fit the far-field background from the scattering signal waveform in the time domain. The algorithm first extracted a short period (120 μs) of the infrared signal waveform around the time of snap-in contact $t_{\mathrm{s}}$ (Supplementary Fig. 11). For a short period (about 10 μs) before $t_{\mathrm{s}}$, the near-field scattering light was considerably enhanced as the tip−sample distance decreases. This short period was defined as the signal region, which corresponded to a short range of tip−sample distance generally less than 5 nm. Prior to this signal region, the infrared signal waveform showed a gradual and approximately linear increase owing to the far-field scattering background. A linear fit of the background was performed for this region and then extrapolated to the signal region. Then, the light scattering of signal region was subtracted by the extrapolated far-field background to obtain the pure near-field responses. The scattering signal after the removal of far-field background was averaged within a defined time window prior to $t_{\mathrm{s}}$ and used as PF-SNOM signal. The average tip−sample distance of the measurement window was calculated with above-described procedure afterward. A real-time program written in LabView (National Instruments) was developed to implement this fast algorithm. This program extracted one value of PF-SNOM signal per specified number of PFT cycles, and PF-SNOM signals from these PFT cycles were averaged per pixel during an AFM scan to form a PF-SNOM image.

### Modified finite dipole model with distance-dependent damping

The finite dipole model was used in the simulation of SiC near-field responses in Supplementary Fig. 9. Details of the finite model were described in Govyadinov et al.^41^. The effective polarizability $\alpha _{{\mathrm{eff}}}$ in finite dipole model is shown below:

9 $$\alpha _{{\mathrm{eff}}} = C\left(2 + \frac{{f_0(d)\beta }}{{1 - f(d)\beta }}\right).$$

The function $f_0(d)$ and $f( d )$ are functions of the tip−sample distance *d*, which are expressed as:

10 $$f_0( d ) = \left(g - \frac{{2d + W_0 + r}}{{2L}}\right)\frac{{\ln ({{4L}}/{{(4d + 2W_0 + r))}}}}{{\ln ({{4L}}/{r})}},$$

11 $$f( d ) = \left(g - \frac{{2d + W_{\mathrm{i}} + r}}{{2L}}\right)\frac{{\ln ({{4L}}/{{4d + 2r}})}}{{\ln ({{4L}}/{r}})},$$

where $W_0 \approx 1.31\;rL/(L + 2r)$, $W_{\mathrm{i}} \approx r/2$, *L* *=* 600 nm and $g = 0.7{\mathrm{e}}^{0.06i}$, and $r$ is the tip radius. These values were obtained from Govyadinov et al.^41^ and were practically invariant for typical s-SNOM tips. In our treatment, a tip−sample distance-dependent phase factor ${\mathrm{e}}^{i\varphi (d)}$ was introduced to $f_0( d )$ and $f( d )$. Similar to Eq. (2), we introduced $f_0\hskip -3pt\prime ( d ) = f_0( d ){\mathrm{e}}^{i\varphi (d)}$ and $f\prime ( d ) = f( d )\;{\mathrm{e}}^{i\varphi (d)}$, where $\varphi ( d ) = a\prime {\mathrm{e}}^{ - d/b\prime }$, where $a\prime$ is the magnitude factor showing strength of damping, and $b\prime$ is the 1/e decay range of damping. In our simulation, ${a\prime}$ = 0.05 and $b\prime = r/10$. The modified effective polarizability of the finite dipole model is then expressed as:

12 $$\alpha _{{\mathrm{eff}}} = C(2 + \frac{{f_0\hskip -3pt\prime ( d )\beta }}{{1 - f^\prime ( d )\beta }}).$$

This $\alpha _{{\mathrm{eff}}}$ was used in the simulation of two-dimensional near-field responses in Fig. 4g, the scattering spectra in Fig. 4h, and the *d* dependence of near-field response in Fig. 4i.

### Measurement of mechanical and electrical properties

The logarithm of modulus and adhesion (Fig. 5b, c) were automatically calculated with PeakForce Tapping QNM software by Bruker Nanoscope. The mechanical measurements were done during the dynamic contact regime in each of the PFT cycles and then were averaged over many cycles for a better SNR. The PF-SNOM signal was extracted prior to the tip−sample contact so that measurement of PF-SNOM signal was independent of the subsequent mechanical and electrical measurements. The electrical contact current measurement in Fig. 5d was performed with a PeakForce-TUNA module of the Multimode AFM in the PFT mode with an electrical bias of 10 V applied between the metallic tip and the sample. The dynamic contact between the tip and the sample allowed passage of electrical current, in addition to simultaneous mechanical and near-field measurements. The multimodal near-field, mechanical, and electrical measurements were done at 1 kHz PFT frequency due to the speed limit of electrical current amplification in the PeakForce-TUNA module.

### Materials

Graphene was purchased from Graphenea, which was produced via CVD on copper films and wet transferred to a SiO_2_/Si substrate. The <100> oriented Si substrate was P-doped. The thickness of SiO_2_ was 300 ± 15 nm. The CVD process occasionally produced double-layer graphene patches at μm scale. CVD-grown BNNT was dispersed in isopropanol and drop-casted on a gold substrate. SiC (4H-SiC) was purchased from University Wafer and directly used. h-BN was purchased from Manchester Nanomaterials, exfoliated with a tape (18074-.50, Semiconductor Equipment Corp.), and transferred to a Si wafer (N-doped, <100> orientation, 1−10 ohms-cm).

### Data availability

The data that support the figures, tables, and plots within this paper, Supplementary Information and other findings of this study are available from the corresponding author upon reasonable request.

## Electronic supplementary material

Supplementary Information
