# Supplementary material for: Tomographic and multimodal scattering-type scanning near-field optical microscopy with peak force tapping mode
Source: Nat Commun. 2018 May 21;9:2005. doi: 10.1038/s41467-018-04403-5 (PMC5962580; doi:10.1038/s41467-018-04403-5)
Supplement: Supplementary file 1 — Supplementary Information [file 41467_2018_4403_MOESM1_ESM.pdf]

Supplementary Information: Tomographic and Multimodal Scattering-Type Scanning Near-field Optical Microscopy with Peak Force Tapping Mode

Haomin Wang[1], Le Wang[1], Devon S. Jakob[1], and Xiaoji G. Xu[1]*

[1]Department of Chemistry, Lehigh University, 6 E Packer Avenue, Bethlehem, PA, 18015, USA

Corresponding Email: xgx214@lehigh.edu

The supplementary information contains 11 figures:

Supplementary Figure 1. **Signal generation mechanism in tapping mode scattering-type scanning near-field microscopy (s-SNOM).**

Supplementary Figure 2. **Discrepancies of tapping mode s-SNOM patterns collected from different orders of lock-in demodulation.**

Supplementary Figure 3. **Differences in spatial patterns of a boron nitride nanotube (BNNT) from different orders of lock-in demodulations in s-SNOM.**

Supplementary Figure 4. **Peak force scattering-type scanning near-field optical microscopy (PF-SNOM) with the synthetic optical holography technique.**

Supplementary Figure 5. **s-SNOM images from tapping mode s-SNOM on the same region of graphene as shown in the main text Fig. 1g.**

Supplementary Figure 6. **s-SNOM images of the BNNT at different infrared frequencies and lock-in demodulation orders.**

Supplementary Figure 7. **Typical spatial resolution of a conventional tapping mode s-SNOM.**

Supplementary Figure 8. **Change of periodicity of phonon polariton (PhP) patterns as tip-sample distance changes**.

Supplementary Figure 9. **Numerical simulation of two-dimensional near-field responses of SiC with the finite dipole model.**

Supplementary Figure 10. **Correlation between measured modulus and PhPs of BNNT simultaneously measured by PF-SNOM.**

Supplementary Figure 11. **Fast method to extract near-field signals in PF-SNOM.**

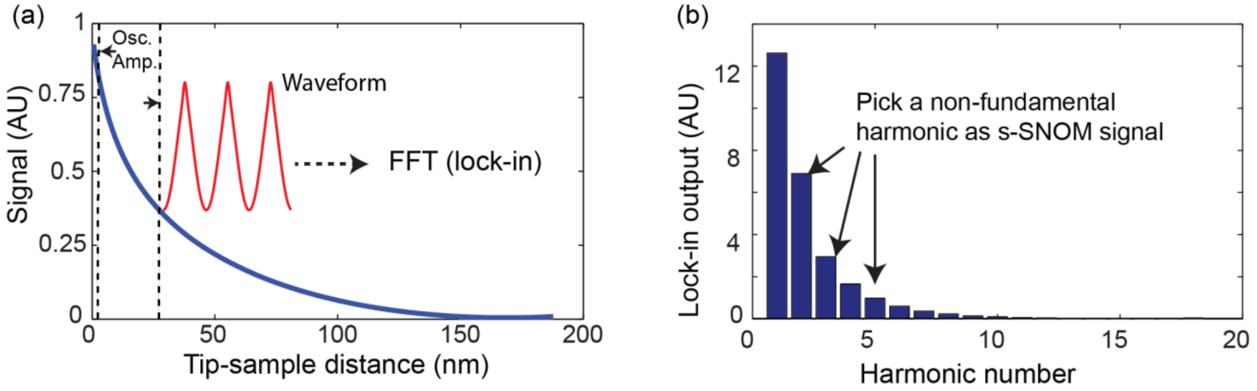

Supplementary Figure 1. **Signal generation mechanism in tapping mode scattering-type scanning near-field microscopy (s-SNOM).** (a) In traditional s-SNOM, the tip is vertically oscillating above the sample surface in tapping mode AFM feedback. The oscillation amplitude of tip is determined by the user through an AFM controller, for example, ~30 nm. When the AFM tip is oscillating close to a sample surface, the tip-sample near-field interaction increases the light scattering signal (schematically shown as the blue curve). As the tip is harmonically oscillating above the sample surface, the amplitude of the scattered optical signal is modulated as the tip oscillates. The vertical dashed lines mark the region of tip-sample distance in which the tip oscillates. As the tip oscillates, the scattering signal from a light detector forms a waveform (red curve) in the time domain. As the near-field response is nonlinear with respect to the tip-sample distance, the waveform contains anharmonicity. Fourier transformation or lock-in demodulation of the waveform is used to reveal the anharmonicity that is characteristic of near-field interactions. (b) Lock-in detection or Fourier analysis typically yields several harmonic components. One of the non-fundamental harmonics (Ω = 2H, 3H, 4H…) from lock-in demodulation is used as the s-SNOM signal. As a result, the s-SNOM signal depends on the choice of lock-in harmonic number and the oscillation amplitude of AFM tip in tapping mode.

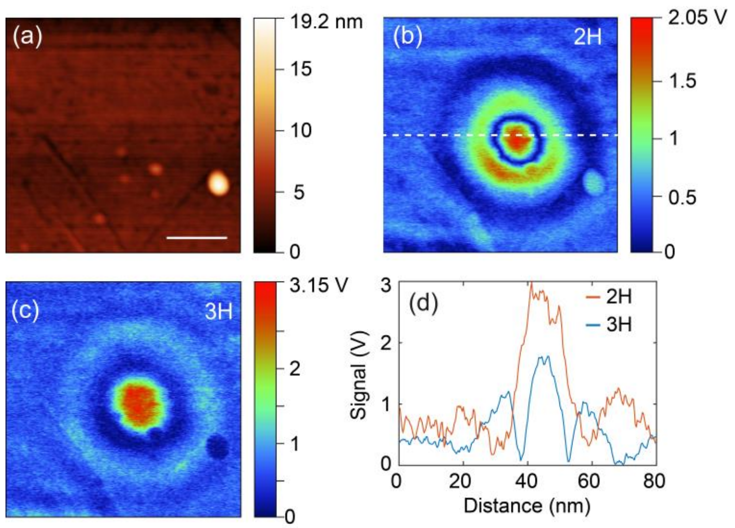

Supplementary Figure 2. **Discrepancies of tapping mode s-SNOM patterns collected from different orders of lock-in demodulation.** Though the tapping mode s-SNOM images from different non-fundamental harmonic demodulations often show similar overall patterns, in certain situations, different harmonic demodulations can exhibit very different patterns. (a) Topography of a bilayer graphene nano-disk covered by monolayer graphene. The scale bar is 200 nm for (a-c). (b) Tapping mode s-SNOM image at 1400 cm^{-1} infrared frequency demodulated at the second harmonic (2H) of the tapping frequency of AFM tip by a multi-channel lock-in amplifier (Zurich Instruments HF2Li). (c) Tapping mode s-SNOM image of the same area and the same infrared frequency from the third harmonic (3H) lock-in demodulation. Considerable differences are observed from (b) and (c), despite that they were collected simultaneously. (d) Extracted signal profiles from (b) and (c), the location of extraction is shown as a white dashed line in (b). While the center of the graphene nano-disk shows a signal maximum in both 2H and 3H, the outer rings are inverted from 2H to 3H images.

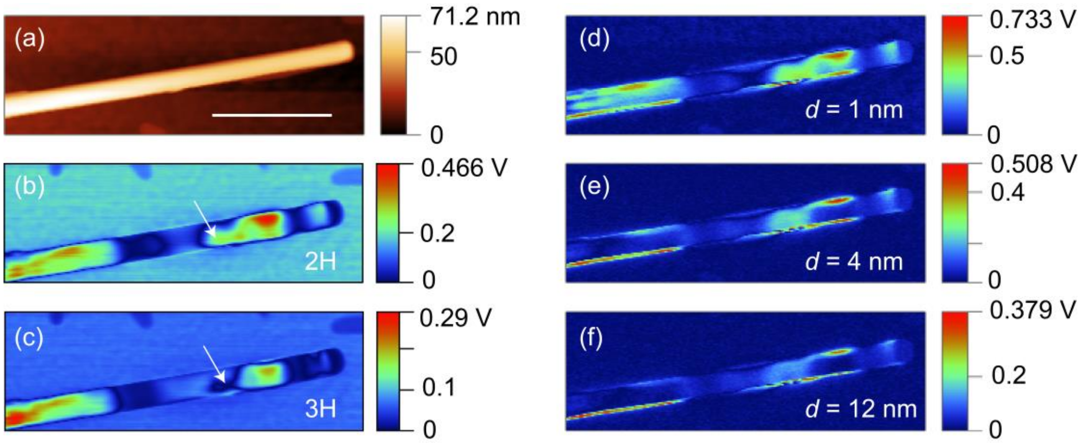

Supplementary Figure 3. **Differences in spatial patterns of a boron nitride nanotube (BNNT) from different orders of lock-in demodulations in s-SNOM.** (a) Topography of a BNNT. The scale bar is 500 nm for all panels. (b) Tapping mode s-SNOM image from the second harmonic lock-in demodulation (2H) of scattering signal. The infrared frequency is 1520 cm^{-1}. (c) s-SNOM image from the third harmonic lock-in demodulation (3H) of the same BNNT at 1520 cm^{-1}. The images in (b) and (c) were collected simultaneously with a multi-channel lock-in amplifier. Considerable differences in spatial patterns are observed. For example, the region marked by white arrows in (b) and (c) shows opposite signal distributions. This is due to the complex generation mechanism of lock-in demodulation. The transduced signal from lock-in demodulation is from the nonlinear change of near-field signals over a range of tip-sample distances, rather than the strength of the near-field signal itself. (d)-(f) PF-SNOM images at the tip-sample distance d = 1, 4 and 12 nm of the same BNNT at the same infrared frequency of 1520 cm^{-1}. PF-SNOM figures show the consistent progression of spatial patterns. Reduced signal amplitude with increasing d can also be observed. This is because PF-SNOM probes vertical near-field response based on the strength of response at each specific tip-sample distance.

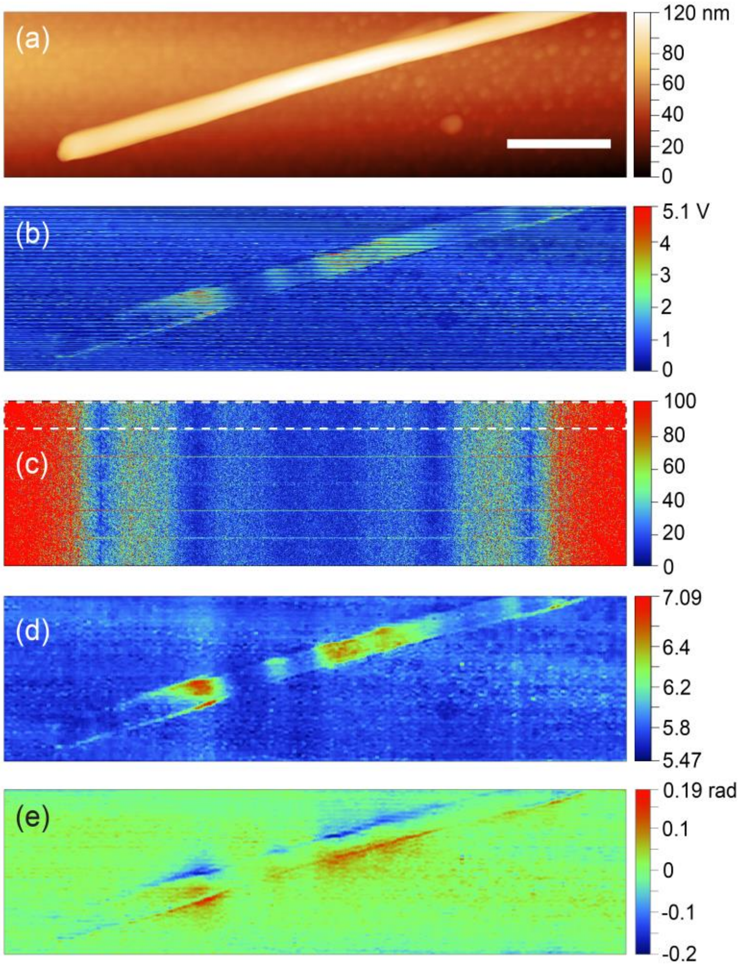

Supplementary Figure 4. **Peak force scattering-type scanning near-field optical microscopy (PF-SNOM) with the synthetic optical holography technique**. (a) Topography of a BNNT obtained in peak force tapping mode. The scale bar is 500 nm for all panels. (b) A 1024×256 pixel PF-SNOM image obtained by the near-field holography method (Schnell et. al[1]), during which the retroreflector of the interferometer was slowly scanned in the range of 40 μm with 0.025 Hz, and quickly back to its original place when 40 μm was reached. 0.15 Hz line scan speed in the AFM was used to acquire this image. (c) 2D Fourier transform of the hologram in (b). Filtering section for inverse fast Fourier transform (FFT) is marked by a 1024×40 white dashed box. (d) Amplitude of the near-field response from the inverse FFT of pixels in the white dashed box in (c). (e) Corresponding phase map obtained from the inverse FFT.

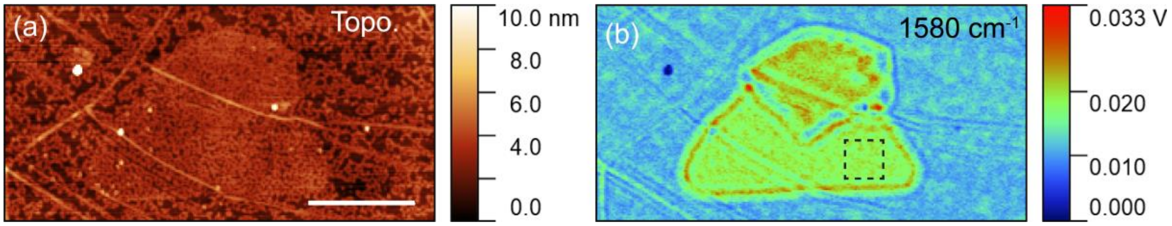

Supplementary Figure 5. **s-SNOM images from tapping mode s-SNOM on the same region of graphene as shown in the main text Fig. 1g.** (a) Topography obtained from the tapping mode. The scale bar is 1 μm for both (a) and (b). (b) Tapping mode s-SNOM image at 1580 cm^{-1} from the third harmonic demodulation. The tapping mode s-SNOM and PFT mode PF-SNOM (see Fig. 1g in the main manuscript) on graphene show overall similar plasmon distribution patterns. Signal-to-noise ratios (SNR) were statistically calculated as the $\frac{average}{rms \cdot \sqrt{T}}$, where *average* is the average value of all signal points and *rms* is the root-mean-square, of the same $0.33 \times 0.33\ \mu m^2$ region in both s-SNOM and PF-SNOM figures, shown as black dashed boxes in (b) and Fig. 1g in the main text, and T is the normalized acquisition time (for s-SNOM, $T = 1/6$; for PF-SNOM, $T = 1$). The calculated SNR for s-SNOM is 40, and 49 for PF-SNOM. Peak-to-peak tapping amplitude was set as 30 nm in the tapping mode.

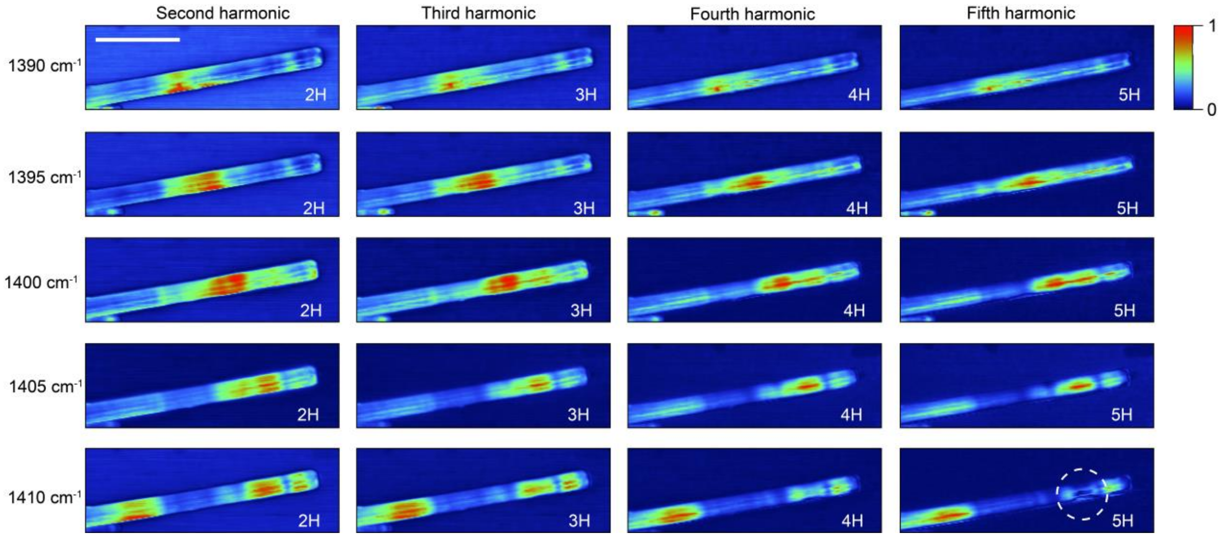

Supplementary Figure 6. **s-SNOM images of the BNNT at different infrared frequencies and lock-in demodulation orders.** The BNNT is the same as the one in Fig. 2 of the main text. s-SNOM provides different near-field images from the 2nd to 5th lock-in demodulations (indicated by 2H to 5H in the figure), the scale bar shown in the first panel is 500 nm, which is the same for all panels. In this measurement, peak-to-peak tapping amplitude was set as 30 nm in the tapping mode. At the same infrared frequency, different harmonic demodulations exhibit different spatial patterns. The 5th harmonic at 1410 cm^{-1}, for example, shows a strange pattern (marked by a white circle) that is difficult to interpret and is likely due to the complicated signal generation mechanism of demodulation of higher harmonics.

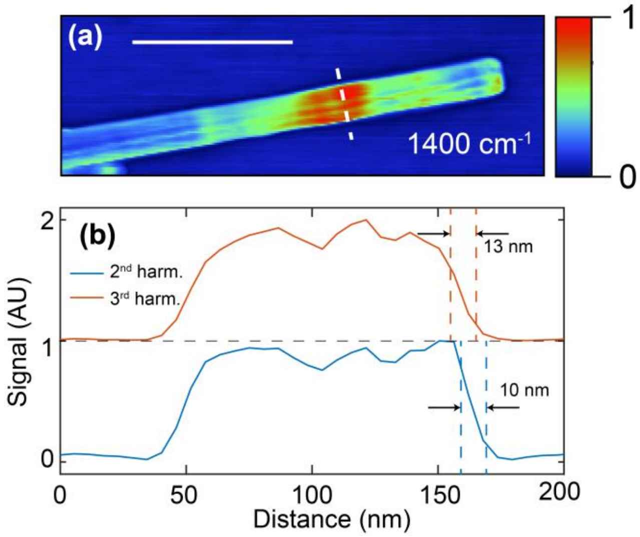

Supplementary Figure 7. **Typical spatial resolution of conventional tapping mode s-SNOM.** (a) Tapping mode s-SNOM image of a BNNT at 1400 cm^{-1} by using the 2H demodulation. The scale bar is 500 nm. (b) Normalized signal profiles obtained from the location indicated by a white dashed line in (a). The blue curve is obtained from the 2H demodulation, while the red curve is obtained from the 3H demodulation. Spatial resolutions of 10 and 13 nm are obtained for the 2H demodulation and the 3H demodulation, respectively. Profiles of the 3H demodulation was offset by 1 AU for the convenience of comparison.

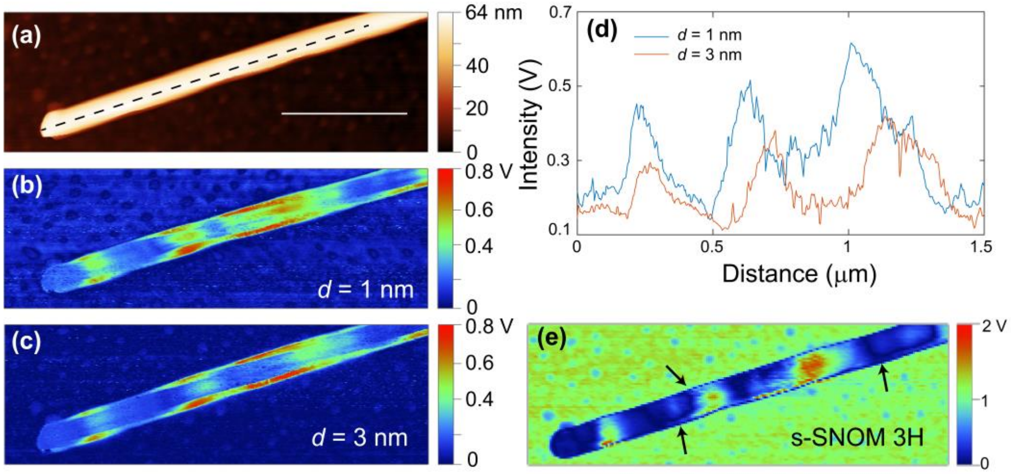

Supplementary Figure 8. **Change of periodicity of phonon polariton (PhP) patterns as tip-sample distance changes**. (a) Topography of a BNNT. The scale bar is 400 nm for (a-c) and (e). (b-c) PF-SNOM images at 1410 cm^{-1} infrared frequency sectioned at tip-sample distance d = 1 and 3 nm. (d) Extracted near-field signal profiles from PF-SNOM images along the BNNT showing the periodicity of PhPs reduces as d decreases. The extraction location is shown as the black dashed line in (a). (e) Reference tapping mode s-SNOM from the 3H demodulation. The image contains signal discontinuities on the BNNT (marked by black arrows) that is a result of signal extraction mechanism. i.e., the 3H harmonic demodulation extracts only a certain degree of signal change of the near-field responses as the tip-sample distance d varies. Signal changes obtained from lock-in demodulation do not guarantee spatial continuity and is not necessarily proportional to the strength of the near-field response.

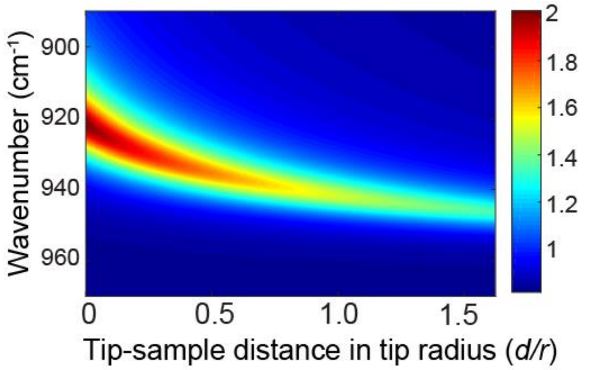

Supplementary Figure 9. **Numerical simulation of two-dimensional near-field responses of SiC with the finite dipole model.** The finite dipole model and dielectric function of SiC are based on literature.[2, 3, 4] A tip radius of 40 nm is used in the simulation. Compared with the simulation of the image dipole model in the main text Fig. 4e, the finite dipole model produces a longer range of near-field interactions and is closer to experimental results in the main text Fig. 4a.

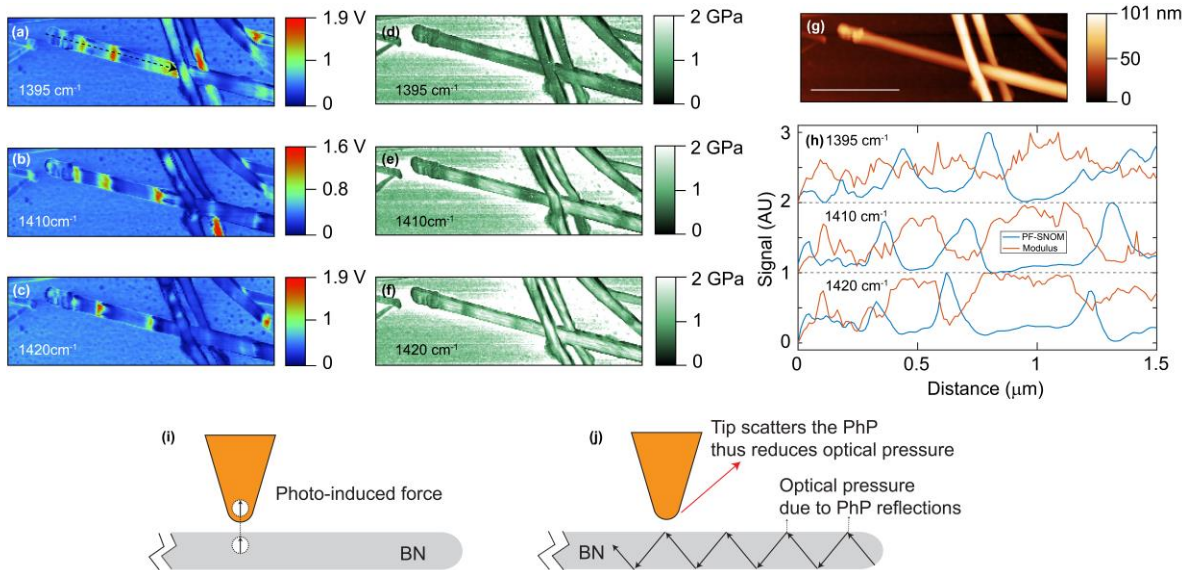

Supplementary Figure 10. **Correlation between measured modulus and PhPs of BNNT simultaneously measured by PF-SNOM**. (a-c) PF-SNOM results on a group of BNNTs at three different infrared frequencies of 1395, 1410 and 1420 cm^{-1}. (d-f) Mechanical modulus obtained simultaneously with (a-c) by the peak force tapping (PFT) mode, which shows opposite contrast to the near-field signals in (a-c). (g) Topography of the same region by the PFT, the scale bar is 1 μm (for a-g). (h) Normalized nearfield signal profiles (blue curves) and modulus profiles (red curves) extracted along the black dashed line in (a). Spatial patterns of near-field signals and modulus both shift with increasing incident light frequency with anti-correlation in the signal contrast. Signals for 1395 and 1410 cm^{-1} were offset by 2 and 1 for the convenience of comparison. The observation indicates that strong PhPs will affect the measured mechanical modulus through the possible presence of optical forces. The origin of the optical force could be the photo-induced force schematically shown in (i),[5] or the release of optical pressure inside the BNNT by the scattering of hyperbolic PhPs within the BNNT by the metallic tip, as illustrated in (j). The photo-induced force in (i) has its origin as following. The induced dipole of the tip generated by the optical field has mechanical force with the induced dipole generated by collective oscillations of PhPs. As the generation of PhPs is spatially dependent on the probing position and its distance to the tube terminal, the photo-induced forces also exhibit spatial variations and are dependent on the infrared frequency. This origin is similar to the photo-induced force that has been exploited in the photo-induced force microscopy.[5, 6] Note that the photo-induced force is attractive and therefore causes the reduction of the measured modulus, which is consistent with our experiment. The second possible mechanism is the optical pressure generated by PhP waves propagating inside the hyperbolic boron nitride. Because the PhPs are reflected and confined by the interior of tube, the reflection of PhP waves passes momentum to the nanotube and increases its modulus. As the metallic tip probing the BNNT from outside, the coupling between the tip and the PhPs leads to the release of PhPs from the interior of BNNT to the outside. Once the PhPs are released from the nanotube through the coupling, the optical pressure generated from PhPs also reduces, and causes a reduction of the measured modulus by peak force tapping. The efficiency of coupling between the tip and the PhPs has spatial dependence and correlates with near-field signals, and then this correlation is passed to the mechanical response from the optical pressure. Because the scattering of PhPs inside the BNNT reduces the optical pressure, the mechanical modulus should also be decreased, which is consistent with our experimental observation. Here, we presented two possible scenarios, and more studies are still needed to determine the exact origin of the correlation between mechanical force and PhPs, which should be the topic of a separate research project.

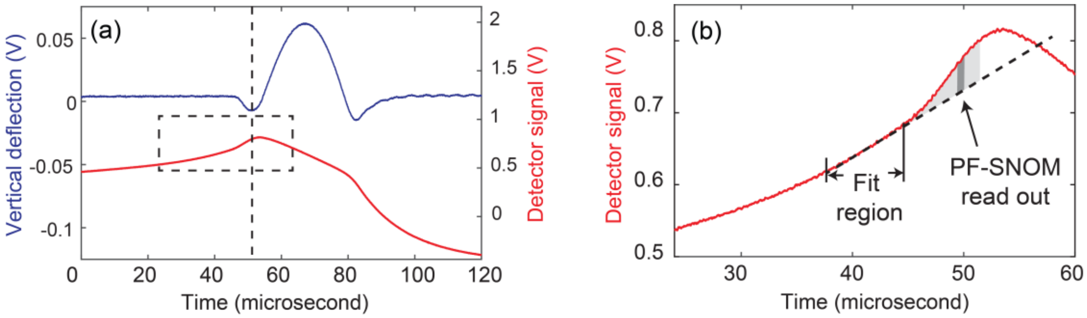

Supplementary Figure 11. **Fast method to extract near-field signals in PF-SNOM.** (a) Simultaneously acquired cantilever deflection signal (blue) and infrared detector signal (red). Snap-in contact is marked by the vertical dashed line. (b) A zoomed-in region of the detector infrared signal from the dashed box in (a) around the time of snap-in contact. A linear background fitting (dashed line) of the trend of far-field scattering is performed and subtracted from the scattering signal to obtain the short-range near-field signal (grey area), a slice of which is used as PF-SNOM signal in imaging mode (dark grey bar).

References:

1. Schnell M., Carney P. S. & Hillenbrand R. Synthetic optical holography for rapid nanoimaging. *Nat. Commun.* **5**, 3499 (2014).

2. Cvitkovic A., Ocelic N. & Hillenbrand R. Analytical model for quantitative prediction of material contrasts in scattering-type near-field optical microscopy. *Opt. Express* **15**, 8550-8565 (2007).

3. Govyadinov A. A. *et al*. Recovery of permittivity and depth from near-field data as a step toward infrared nanotomography. *ACS Nano* **8**, 69116921 (2014).

4. Taubner T., Keilmann F. & Hillenbrand R. Nanomechanical resonance tuning and phase effects in optical near-field interaction. *Nano Lett.* **4**, 1669-1672 (2004).

5. Jahng J. *et al*. Gradient and scattering forces in photoinduced force microscopy. *Phys. Rev. B* **90**, 155417 (2014).

6. Nowak D. *et al*. Nanoscale chemical imaging by photoinduced force microscopy. *Sci. Adv.* **2**, e1501571 (2016).
